# Trial watch: antibody-drug conjugates in cancer therapy

**DOI:** 10.1080/2162402X.2026.2667160

**Published:** 2026-05-03

**Authors:** Flora Doffe, Xiaolian Deng, Zhe Shen, Enfu Xue, Kristine Schauer, Guido Kroemer, Oliver Kepp

**Affiliations:** a Université Paris Cité, Sorbonne Université, Inserm, Centre de Recherche des Cordeliers, Equipe labellisée par la Ligue contre le cancer, Institut Universitaire de France, Paris, France; b Université Paris-Saclay, INSERM US23/CNRS UAR 3655, Metabolomics and Cell Biology Platforms, Gustave Roussy, Villejuif, France; c Cell Biology of Organelle Networks Team, Tumor Cell Dynamics Unit, Inserm U1279 Gustave Roussy Institute, Université Paris-Saclay, Villejuif, France; d Centre National de la Recherche Scientifique (CNRS), Paris, France; e Institut du Cancer Paris CARPEM, Department of Biology, Hôpital Européen Georges Pompidou, AP-HP, Paris, France

**Keywords:** Cancer immunotherapy, bystander effect, topoisomerase I inhibitor, immunogenic cell death

## Abstract

Antibody-drug conjugates (ADCs) combine the target specificity of monoclonal antibodies with the cytotoxic potency of small-molecule payloads. In recent years ADCs have emerged as a clinically validated component of modern precision oncology. To date, more than a dozen ADCs have received FDA approval for oncologic indications, with additional agents approved regionally and hundreds of ADC-based regimens in clinical development. Collectively, these therapies have demonstrated clinical benefit across hematologic malignancies and solid tumors, including significant overall survival improvements in advanced phase clinical trials. In this Trial Watch, we provide an overview on available ADCs from early preclinical development to current clinical applications. We also summarize design principles underpinning clinically successful ADCs, including epitope targeting, linker chemistry, payload toxicity and drug-to-antibody ratio, and discuss how these features can influence pharmacokinetics, intracellular trafficking, bystander effect and toxicity. Finally, we discuss results from advanced-stage clinical trials and approved agents to define future directions.

## Introduction

The concept of selectively targeting malignant cells while sparing normal tissues has long been a central objective of cancer therapy. Antibody-drug conjugates (ADCs) represent the clinical realization of this principle, combining the antigen specificity of monoclonal antibodies with the cytotoxic potency of small-molecule payloads thus improving therapeutic outcomes relative to conventional, untargeted chemotherapy.^1-4^

The development of ADCs has progressed through distinct phases, which is reflected in the payload and target diversity of agents clinically approved today^5^ (Table 1). The first regulatory approval of an ADC, *gemtuzumab ozogamicin* for acute myeloid leukemia (AML) in 2000,^6^ established proof of concept, and its subsequent withdrawal and later reapproval, attributed to limitations in linker stability, dosing and patient selection, also highlighted the challenges of optimizing multicomponent biologics as well as trial design.^7^ Nevertheless, subsequent approvals of *brentuximab vedotin*^8^^,^^9^ and *trastuzumab emtansine*^10^ demonstrated that ADCs could deliver durable clinical benefit in both hematologic malignancies and solid tumors when equipped with appropriate linker-payload combinations and directed against validated targets.^11-14^ Together, these outcomes elevated ADCs from a mere experimental approach to a viable therapeutic modality. Since then the ADC landscape has exponentially expanded, with approvals spanning multiple tumor types (Table 1). Next-generation ADCs, including *trastuzumab deruxtecan*, *sacituzumab govitecan* and *tisotumab vedotin*, incorporate advanced linker chemistry, increased drug-to-antibody ratio and payloads capable of bystander killing.^15-18^ In sum these features enable ADC activity in tumors with heterogeneous antigen expression even outside narrowly defined biomarker-positive populations. More recent regulatory approvals, such as those for *disitamab vedotin* (currently regionally approved), and regionally approved next-generation ADCs with novel exatecan-derived payloads inlcuding *trastuzumab rezetecan* (SHR9265-based ADC), and *sacituzumab tirumotecan* (KL610023-based ADC), further illustrate the maturation of the ADC field and the diversification of design strategies.

**Table 1.** List of clinically approved antibody drug conjugates in oncology.

ADC	Antibody target	Payload (Class)	Linker	Approved indication(s)	Year/Region
***Gemtuzumab ozogamicin*** **(Mylotarg®)**	CD33	Calicheamicin (DNA-damaging)	Acid-labile	Acute myeloid leukemia	2000/FDA
***Brentuximab vedotin*** **(Adcetris®)**	CD30	MMAE (microtubule inhibitor)	Protease-cleavable	Hodgkin lymphoma, T cell lymphomas	2011/FDA
***Trastuzumab emtansine*** **(Kadcyla®)**	HER2	DM1 (microtubule inhibitor)	Non-cleavable	HER2^+^ breast cancer	2013/FDA
***Inotuzumab ozogamicin*** **(Besponsa®)**	CD22	Calicheamicin	Acid-labile	B cell acute lymphoblastic leukemia	2017/FDA
***Polatuzumab vedotin*** **(Polivy®)**	CD79b	MMAE (microtubule inhibitor)	Protease-cleavable	Diffuse large B cell lymphoma	2019/FDA
***Enfortumab vedotin*** **(Padcev®)**	Nectin-4	MMAE (microtubule inhibitor)	Protease-cleavable	Urothelial carcinoma	2019/FDA
***Trastuzumab deruxtecan*** **(Enhertu®)**	HER2	Deruxtecan (topoisomerase I inhibitor)	Enzyme-cleavable	Breast, gastric, lung cancers	2019/PMDA
***Sacituzumab govitecan*** **(Trodelvy®)**	TROP2	SN-38 (topoisomerase I inhibitor)	Hydrolysable	Breast, urothelial cancer	2020/FDA
***Tisotumab vedotin*** **(Tivdak®)**	F3 (CD142)	MMAE (microtubule inhibitor)	Protease-cleavable	Cervical cancer	2021/FDA
***Loncastuximab tesirine*** **(Zynlonta®)**	CD19	PBD dimer (DNA crosslinker)	Protease-cleavable	Diffuse large B cell lymphoma	2021/FDA
***Belantamab mafodotin*** **(Blenrep®)**	BCMA	MMAF (microtubule inhibitor)	Non-cleavable	Multiple myeloma	2020/EMA
***Trastuzumab duocarmazine***	HER2	Duocarmycin (DNA alkylator)	Cleavable	Breast cancer	2022/EMA
***Disitamab vedotin***	HER2	MMAE (microtubule inhibitor)	Protease-cleavable	Gastric, urothelial cancer	2021/China
***Mirvetuximab soravtansine*** **(Elahere®)**	FOLR1	DM4 (microtubule inhibitor)	Cleavable	Ovarian cancer	2022/FDA
***Trastuzumab rezetecan*** **(SHR9265)**	HER2	SHR9265 (Rezetecan, topoisomerase I inhibitor)	Cleavable	Gastric cancer	2021/China
***Sacituzumab tirumotecan*** **(KL610023)**	TROP2	KL610023 (topoisomerase I inhibitor)	Cleavable	Solid tumors	2022/China
***Datopotamab deruxtecan*** **(Datroway®)**	TROP2	DXd (topoisomerase I inhibitor)	Enzyme-cleavable	Breast cancer	2024-2025/JP-US

Table includes antibody-drug conjugates (ADC) approved by at least one major regulatory authority (FDA, EMA, PMDA, or NMPA). Abbreviations: DXd, deruxtecan; MMAE, monomethyl auristatin E; MMAF, monomethyl auristatin F; PBD, pyrrolobenzodiazepine. ADCs are referred to using internationally recognized generic names (INN) where available; for regionally approved agents or investigational compounds, commonly used translational or clinical trial names are provided, with payload designations indicated where appropriate. Status 02/2026.

Collectively, the approved ADCs summarized in Table 1 highlight shared characteristics that have shaped clinical success including the convergence on a set of clinically actionable targets, the repeated use of payloads from the class of microtubule and topoisomerase I inhibitors, and improved linker chemistry for controlled intracellular drug delivery. Together these characteristics have enabled ADCs to move from late-line salvage therapy into earlier treatment settings and combination regimens. In this Trial Watch, we review molecular characteristics, preclinical strategies and clinical development trajectories that underpin ADCs currently approved for oncology (Table 1) and synthesize emerging data from late-stage clinical trials (Table 2) that define the present and future role of ADCs in clinical oncology.

**Table 2.** Selected phase III-IV antibody-drug conjugate clinical trials.

ADC	Target	Payload	Phase	Indication/line of therapy/co-medication	NCT ID	Trial status
***Belantamab mafodotin***	BCMA	MMAF	III	Relapsed/refractory multiple myeloma (≥2L); combination therapy	NCT04162210	Active, not recruiting
***Belantamab mafodotin***	BCMA	MMAF	III	Relapsed/refractory multiple myeloma (≥2L); combination therapy	NCT04246047	Active, not recruiting
***Belantamab mafodotin***	BCMA	MMAF	III	Relapsed/refractory multiple myeloma (≥2L); combination therapy	NCT04484623	Active, not recruiting
***Brentuximab vedotin***	CD30	MMAE	III	Newly diagnosed classical Hodgkin lymphoma; frontline; with AVD	NCT03907488	Active, not recruiting
***Datopotamab deruxtecan***	TROP2	DXd	III	Metastatic breast cancer; previously treated; monotherapy	NCT05374512	Active, not recruiting
***Datopotamab deruxtecan***	TROP2	DXd	III	HR^+^/HER2^-^ metastatic breast cancer; previously treated; monotherapy	NCT05104866	Active, not recruiting
***Datopotamab deruxtecan***	TROP2	DXd	III	Metastatic breast cancer; previously treated; with durvalumab	NCT06103864	Recruiting
***Datopotamab deruxtecan***	TROP2	DXd	III	Metastatic breast cancer; previously treated; ± durvalumab	NCT05629585	Active, not recruiting
***Enfortumab vedotin***	Nectin-4	MMAE	IV	Muscle-invasive bladder cancer; perioperative; with pembrolizumab	NCT06764095	Recruiting
***Enfortumab vedotin***	Nectin-4	MMAE	III	Untreated locally advanced/metastatic urothelial carcinoma; first-line; with pembrolizumab	NCT04223856	Active, not recruiting
***Gemtuzumab Ozogamicin***	CD33	Calicheamicin	III	AML; first-line; monotherapy	NCT04168502	Recruiting
***Inotuzumab ozogamicin***	CD22	Calicheamicin	III	Relapsed/refractory B-ALL; monotherapy	NCT03677596	Active, not recruiting
***Loncastuximab tesirine***	CD19	PBD dimer	III	Relapsed/refractory DLBCL (≥2L); monotherapy	NCT04384484	Active, not recruiting
***Patritumab deruxtecan***	HER3	DXd	III	Metastatic NSCLC; previously treated; monotherapy	NCT05338970	Active, not recruiting
***Sacituzumab govitecan***	TROP2	SN-38	III	Metastatic or refractory urothelial carcinoma (including stage IV bladder cancer), monotherapy	NCT04579224	Recruiting
***Sacituzumab govitecan***	TROP2	SN-38	III	Triple negative or metastatic breast cancer; monotherapy	NCT05552001	Recruiting
***Sacituzumab govitecan***	TROP2	SN-38	III	Triple negative breast cancer; monotherapy	NCT06081244	Recruiting
***Sacituzumab govitecan***	TROP2	SN-38	III	Resectable non-small cell lung carcinoma (monotherapy).	NCT06431633	Recruiting
***Sacituzumab govitecan***	TROP2	SN-38	III	Urothelial Carcinoma; monotherapy	NCT06524544	Recruiting
***Trastuzumab deruxtecan***	HER2	DXd	III	HER2-low unresectable/metastatic breast cancer; previously treated; monotherapy	NCT03734029	Active, not recruiting
***Trastuzumab deruxtecan***	HER2	DXd	IV	HER2^+^ solid tumors; post-marketing safety; monotherapy	NCT06429761	Recruiting
***Trastuzumab deruxtecan***	HER2	DXd	IV	Anatomic Stage IV Breast Cancer; Metastatic Breast Carcinoma; Metastatic Malignant Neoplasm in the Brain; monotherapy	NCT05376878	Recruiting

Abbreviations: ADC, Antibody drug conjugate; AML, acute myeloid leukemia; B-ALL, B cell acute lymphoblastic leukemia; DLBCL, diffuse large B cell lymphoma; ICI, immune checkpoint inhibitor; NSCLC, non-small-cell lung cancer; RT, radiotherapy; TMZ, temozolomide. ADCs are referred to using internationally recognized generic names (INN) where available; for regionally approved agents or investigational compounds, commonly used translational or clinical trial names are provided, with payload designations indicated where appropriate. Status 02/2026.

### ADC molecular design and mechanism of action

The clinical performance of modern ADCs is determined by the integrated action of their antibody, linker and payload components with therapeutic efficacy requiring balanced target selectivity, drug delivery efficacy, cytotoxic potency, as well as systemic tolerability.^19^^,^^20^ Most clinically advanced ADCs employ IgG1 monoclonal antibodies selected for high-affinity binding to tumor-associated antigens preferentially expressed on malignant cells, that are extracellularly accessible and capable of efficient payload internalization upon antibody engagement.^21^ Clinically validated ADC targets, including HER2, TROP2 and Nectin-4 for solid tumors, as well as CD30, CD33 and BCMA for hematologic malignancies, reflect a convergence on antigens with favorable expression profiles and internalization kinetics.^22-25^ In addition to serving as a targeting vehicle, the antibody component may contribute to antitumor activity through immune effector mechanisms such as antibody-dependent cellular cytotoxicity (ADCC) or complement activation.^26^^,^^27^ However, conjugation of cytotoxic payloads can alter Fc-mediated functions, and the relative contribution of immune effector activity versus payload-mediated cytotoxicity varies among ADCs and remains an area of active investigation.^28^^,^^29^

The linker connecting the antibody and payload is a critical determinant of ADC stability, pharmacokinetics and therapeutic efficacy.^30^ Accordingly, linkers must remain stable in systemic circulation to prevent premature payload release while enabling efficient drug liberation within target cells. Two broad classes of linkers, cleavable and non-cleavable, have been clinically validated.^31^ Cleavable linkers exploit intracellular conditions distinct from plasma, including acidic lysosomal pH, protease activity or a reducing intracellular environment. These linkers release free, often membrane-permeable payloads and are frequently associated with bystander antitumor activity.^32^ Clinically successful examples of ADCs employing cleavable linkers include *enfortumab vedotin*, *brentuximab vedotin* and *sacituzumab govitecan*. By contrast, non-cleavable linkers, typically thioether-based, require complete lysosomal degradation of the antibody to generate an active payload-amino acid conjugate. Although this strategy limits bystander effects, it confers superior plasma stability and more predictable pharmacokinetics such as in *trastuzumab emtansine* and *inotuzumab ozogamicin* (Table 1).

ADC payloads are typically highly potent cytotoxic agents, with intrinsic activities in the picomolar to low-nanomolar range, to compensate for limited antigen density and incomplete intracellular drug delivery.^27^^,^^31^ Most clinically successful payloads fall into two mechanistic classes: microtubule inhibitors and DNA-damaging agents.^33^^,^^34^ Microtubule-disrupting payloads, including auristatins (MMAE, MMAF) and maytansinoids (DM1, DM4), interfere with tubulin dynamics and induce mitotic arrest. These payloads are cell cycle-dependent and have demonstrated strong activity in hematologic malignancies and selected solid tumors, although peripheral neuropathy can be dose-limiting.^30^ DNA-damaging payloads, including topoisomerase I inhibitors such as deruxtecan and exatecan derivatives (including rezetecan (SHR9265) and tirumotecan (KL610023)) and alkylating agents such as pyrrolobenzodiazepines (PBD) or duocarmycins, act independently of cell division and often generate membrane-permeable metabolites,^35^ with DXd- and SN-38-based ADCs enabling substantially higher drug-to-antibody ratio (DAR) than earlier MMAE or PBD constructs.^36-38^ These properties confer robust bystander activity and have been associated with broad efficacy across tumors with heterogeneous antigen expression in multiple late-stage trials.^39^ Conventional conjugation methods generate heterogeneous ADC preparations with DAR values typically ranging between 0 and 8. Of note higher DAR values can enhance cytotoxic potency but are also frequently associated with increased hydrophobicity, accelerated clearance and aggregation, thereby narrowing the therapeutic window.^40^ Clinical experience indicates that, depending on the payload, moderate DAR values provide an optimal balance between efficacy and tolerability for many ADCs.^41^ Collectively, these design principles underscore that ADC efficacy and safety emerge from coordinated optimization of antibody, linker, payload and DAR rather than from any single feature.^27^^,^^42^^,^^43^ Lessons learned from approved ADCs and late-stage failures have directly informed the design of next-generation constructs and provide a mechanistic foundation for the clinical outcomes summarized in Table 2.

### Cellular uptake, intracellular trafficking, and immunogenic cell death

The therapeutic activity of ADCs depends on a multistep intracellular process encompassing antigen binding, receptor-mediated internalization, endosomal trafficking, lysosomal processing, payload release and engagement of intracellular targets.^2^^,^^44^ Lack of efficiency at any of those steps can substantially limit antitumor activity and contribute to primary or acquired resistance.^45^ Following antigen binding, ADC**-**receptor complexes are internalized predominantly via clathrin-mediated endocytosis, although alternative pathways may operate depending on the target receptor.^46^ Internalized complexes traffic through early and late endosomal compartments to lysosomes, facilitating linker cleavage or antibody degradation, thus enabling payload release. Efficient lysosomal delivery is critical, as defects in endosomal maturation, altered lysosomal pH, or impaired protease activity can attenuate payload liberation and reduce cytotoxic efficacy.

A key differentiating feature among ADC classes is their ability to mediate bystander killing of neighboring tumor cells lacking (sufficient) target antigen expression. This property is primarily determined by linker chemistry and payload membrane permeability. ADCs releasing membrane-permeable payloads, such as MMAE or topoisomerase I inhibitors including SN-38 and deruxtecan, can induce cytotoxic effects beyond antigen-positive cells, whereas hydrophilic payloads or non-cleavable linker designs generally blunt bystander activity.^47^ The clinical relevance of bystander effects is context-dependent. In solid tumors with heterogeneous antigen expression, bystander killing can mitigate clonal escape and enhance efficacy, as suggested by the broad activity of certain topoisomerase I**-**based ADCs. Conversely, excessive bystander activity may increase off-target toxicity if payload release occurs in normal tissues or within the tumor microenvironment, underscoring the need to balance potency and safety through rational ADC design and extensive preclinical testing.^27^ Notably, agents such as *sacituzumab govitecan* and *trastuzumab deruxtecan* have been associated with higher rates of adverse events, which may in part reflect their pronounced bystander effects.^48^^,^^49^

Beyond direct cytotoxicity and bystander killing, accumulating evidence indicates that ADC-mediated intracellular processing can engage immune-dependent mechanisms of tumor control.^50^ In particular, selected ADCs have been shown to induce immunogenic cell death (ICD), thereby coupling tumor cell killing to activation of antitumor immunity.^51-54^ ICD is a regulated form of cell death characterized by the coordinated emission of danger-associated molecular patterns, including calreticulin exposure, ATP secretion, and HMGB1 release, which collectively promote dendritic cell recruitment, antigen presentation and T cell priming.^52^ Mechanistic studies further demonstrated that several cytotoxic mechanisms relevant to modern ADC payloads, particularly DNA damage and transcriptional inhibition, can be potent triggers of ICD.^55-58^ Inhibition of transcription leads to unresolved endoplasmic reticulum stress, activation of the integrated stress response, and induction of type I interferon signaling, all of which contribute to danger signal emission and immune activation.^59^ These pathways are especially relevant to ADCs carrying topoisomerase I inhibitor payloads, which simultaneously induce DNA strand breaks and suppress transcriptional elongation, thereby amplifying ICD-associated stress responses. Consistent with these principles, recent preclinical ADC-focused studies demonstrate that topoisomerase I**-**based ADCs induce canonical ICD hallmarks, including calreticulin exposure and HMGB1 release, and elicit immune-dependent antitumor effects in vivo.^48^^,^^60^ DXd-based ADCs have been shown to enhance dendritic cell activation and antigen cross-presentation, providing a mechanistic rationale for the clinically observed synergy between ADCs and immune checkpoint blockade.^56-58^ These observations align with late-stage clinical data for *trastuzumab deruxtecan,*^49^^,^^61-63^ in which durable responses and activity across immunologically diverse tumors suggest an immune-modulatory component beyond direct cytotoxicity. Additional support comes from combination and disease-specific studies. In the BEGONIA trial in triple-negative breast cancer, *trastuzumab deruxtecan* combined with immune checkpoint blockade demonstrated encouraging response rates in a tumor type typically considered immunologically challenging, further supporting the hypothesis that ADC-mediated cytotoxicity may enhance tumor immunogenicity.^64^ Similarly, in gastric cancer, results from DESTINY-Gastric03 and DESTINY-Gastric05 trials indicate clinically meaningful activity of *trastuzumab deruxtecan* in heavily pretreated and heterogeneous patient populations, further highlighting its efficacy beyond narrowly defined biomarker-positive settings.^16^

That said, there is also evidence that microtubular inhibitors can trigger ICD,^65-68^ suggesting that the vast majority, if not all, clinically approved ADCs might be endowed with ICD-inducing properties.

Collectively, these data support a model in which ADC-induced transcriptional stress, DNA damage and intrinsic cytotoxicity converge to promote ICD, reinforcing the rationale for combining selected ADCs with immunotherapies (such as T cell targeted immune checkpoint inhibition (ICI)) and for prioritizing payload classes capable of engaging immune-dependent mechanisms of tumor control as ADCs move into earlier disease settings. More broadly, these observations underscore that ADC efficacy is governed by an integrated chain of intracellular events rather than antigen expression alone. Understanding cellular uptake, trafficking and resistance mechanisms has direct implications for clinical development, informing payload selection, linker design, sequencing strategies and rational combination approaches required to sustain the overall survival benefits observed in late-stage clinical trials.^69^

### Preclinical development and animal models

Preclinical development of ADCs is centered on rigorous target validation, as accumulated clinical experience has consistently demonstrated that antigen selection is a primary determinant of late-stage success. Canonical examples include HER2-, TROP2-, and Nectin-4-targeting ADCs, where high tumor expression, efficient internalization and limited normal tissue distribution translated into durable clinical benefit. By contrast, targets such as CD44v6 or mesothelin, despite strong preclinical rationale, have exhibited inconsistent clinical performance due in part to heterogeneous expression and normal tissue liabilities. Candidate targets are therefore systematically evaluated for tumor-selective expression, cell-surface accessibility, internalization capacity and functional relevance to tumor growth or survival.^27^^,^^70^^,^^71^

Immunohistochemistry and quantitative flow cytometry are routinely applied across large tumor microarrays and normal tissue panels to define expression prevalence and intensity. For example, HER2 expression thresholds used in preclinical development of *trastuzumab deruxtecan* were benchmarked against clinical IHC scoring systems, while TROP2 ADC programs used quantitative flow cytometry to stratify cell lines into high-, intermediate- and low-expressing models.^36^^,^^72^ Target-expression mapping has repeatedly predicted both success and failure. A clinical example is CD44v6, where broad expression on skin keratinocytes translated into severe skin toxicity (including a fatal event) with *bivatuzumab mertansine*, leading to termination of development, a prototype example of how normal-tissue antigen mapping can forecast on-target/off-tumor liabilities.^73^ In contrast, targets such as HER2 and Nectin-4 benefited from strong tumor expression and efficient internalization, enabling clinically transformative ADCs (e.g., *trastuzumab deruxtecan*; *enfortumab vedotin* in urothelial carcinoma), illustrating how target biology and delivery compatibility can align with clinical benefit.^74^^,^^75^

Furthermore, studies using isogenic cell systems with tunable antigen expression have shown that MMAE-based ADCs often require high antigen expression to achieve robust cytotoxicity, whereas topoisomerase I-based ADCs retain activity at substantially lower antigen densities due to membrane-permeable payloads and bystander killing.^76^ Thus, comparative studies in HER2-low versus HER2-high breast cancer cell lines demonstrated that *trastuzumab deruxtecan* maintained activity where *trastuzumab emtansine* was largely inactive, highlighting how payload class modulates antigen-density requirements. In vitro cell line models provide initial proof-of-concept and mechanistic insight into ADC behavior with cytotoxicity assays typically being conducted across panels of antigen-positive and antigen-negative cell lines. Thus to establish specificity and therapeutic index, matched pairs such as HER2⁺ SK-BR-3 versus HER2⁻ MDA-MB-231, or TROP2⁺ HCC1806 versus TROP2-low MCF-7 have been used.^72^^,^^77^ Time-course experiments combined with confocal microscopy or live-cell imaging elucidate internalization pathways, intracellular trafficking, lysosomal delivery, and linker cleavage.^78^ Cathepsin-cleavable linker dependence has been demonstrated in lysosomal protease-deficient cell models, where impaired linker processing attenuates payload release and cytotoxicity.^76^ Moreover, internalization kinetics are commonly assessed using fluorescently labeled antibodies in HER2⁺ breast cancer cell lines (e.g., SK-BR-3, BT-474), TROP2⁺ epithelial tumor lines (e.g., MDA-MB-231, HCC1806), or Nectin-4⁺ urothelial models, with rapid and sustained internalization correlating with superior ADC activity.^70^^,^^79^

However, conventional two-dimensional monolayer cultures frequently overestimate ADC potency by failing to capture architectural constraints on tumor penetration. Three-dimensional tumor spheroids and patient-derived organoids more closely recapitulate diffusion gradients, hypoxia, and heterogeneous antigen expression. Studies using HER2⁺ spheroids and colorectal cancer organoids have shown marked reductions in ADC efficacy relative to 2D cultures, particularly for ADCs with limited bystander effect, whereas DXd- and SN-38-based ADCs demonstrate superior penetration and retained activity in these systems.^36^^,^^80^^,^^81^ Such models have proven predictive of clinical response heterogeneity and resistance emergence. Mouse xenograft models remain the cornerstone of in vivo ADC evaluation. Human tumor cell line-derived xenografts (e.g., BT-474, NCI-N87, MDA-MB-231) and patient-derived xenografts are used to assess tumor growth inhibition, dose-response relationships, and durability of response. These models were instrumental in differentiating the in vivo efficacy of *trastuzumab deruxtecan* from earlier HER2 ADCs and in demonstrating dose-dependent tumor regressions with *sacituzumab govitecan* across multiple epithelial tumor models. Importantly, efficacy correlates strongly with maintenance of clinically relevant antigen expression levels in vivo.

A major limitation of standard xenograft models is the lack of cross-reactivity between human-specific ADCs and murine antigens, which could mask on-target toxicity and underestimates normal tissue exposure. To address this, surrogate ADCs recognizing murine antigens, transgenic mice expressing human target antigens, or humanized mouse models are frequently employed to evaluate safety, biodistribution, and target-mediated clearance.^82^^,^^83^ These approaches have been critical for identifying dose-limiting toxicities associated with targets such as HER2 and TROP2. In addition, syngeneic tumor models in immunocompetent mice enable evaluation of ADC-immune system interactions and rational combination strategies. Using murine surrogates of clinically relevant ADCs, studies in models such as MC38 or EMT6 have shown that certain ADCs induce ICD, promote antigen release, enhance dendritic cell maturation, and increase CD8⁺ T cell infiltration. These findings provided mechanistic support for ADC-ICI combinations that later demonstrated clinical efficacy, including *enfortumab vedotin* plus *pembrolizumab* and *trastuzumab deruxtecan*-based regimens.^49^^,^^57^^,^^84^

Preclinical pharmacokinetic studies characterize systemic exposure, clearance, and tissue distribution of ADCs, including total antibody, conjugated antibody, and free payload concentrations. Comparative studies using DAR-engineered variants have shown that increasing DAR from 4 to 8 can significantly accelerate clearance and increase liver uptake, particularly for hydrophobic payloads such as MMAE, whereas more hydrophilic topoisomerase I payloads exhibit improved tolerability at higher DARs.^20^^,^^42^ Linker stability is assessed by monitoring divergence between total and conjugated antibody exposure, with premature payload release correlating with systemic toxicity and reduced tumor delivery.^85^ Moreover, biodistribution studies using radiolabeled ADCs or quantitative tissue sampling consistently demonstrate preferential tumor accumulation alongside substantial uptake in liver, spleen, and other reticuloendothelial organs. These data have been central to explaining exposure-limiting toxicities observed clinically despite favorable tumor responses and to guiding dose and schedule selection.^86^

Collectively, preclinical ADC models provide essential mechanistic and translational insight, but clinical experience underscores that preclinical efficacy alone is insufficient to predict success. The principal value of preclinical ADC development lies in identifying liabilities, such as inadequate target density, penetration limitations, unstable linkers, or unfavorable pharmacokinetics, early in development, thereby increasing the likelihood of durable efficacy and acceptable safety in Phase III-IV clinical trials.

### Clinical development of antibody-drug conjugates

Phase I studies of ADCs are designed to establish safety, define the recommended phase II dose, and characterize the pharmacokinetics of the conjugate, antibody, and released payload. Because late-stage clinical outcomes are strongly influenced by payload class and linker stability, early-phase trials place particular emphasis on identifying dose-limiting toxicities and defining exposure-toxicity relationships. Across approved ADCs, common early toxicities include hematologic suppression, peripheral neuropathy associated with microtubule inhibitor-based payloads and hepatotoxicity. These effects reflect both payload-related toxicities and off-target uptake.

Early-phase clinical studies illustrate how modern ADC development is converging on validated targets. At the same time payload strategies and clinical positioning are diversified. Topoisomerase I-based payloads remain central but they are increasingly deployed in rational combinations. This trend is exemplified by the Phase 1b/2 modular study of *patritumab deruxtecan* combined with *olaparib* in metastatic HER2-positive breast cancer (NCT06298084), reflecting growing confidence in tolerability and combination compatibility. Similarly, HER2-directed *disitamab vedotin* is being evaluated beyond traditional indications, including in locally advanced and metastatic ERBB2-mutant non-small cell lung cancer in an early Phase 2 combination study (NCT05847764). At the same time, first-in-human programs such as the Phase 1/2 study of ADCE-D01 in metastatic soft-tissue sarcoma (NCT06797999) highlight continued experimentation with novel ADC architectures and payloads in biologically heterogeneous tumors.^87^ Collectively, these early-phase trials underscore a shift toward leveraging established targets with improved molecular design. This evolution is enabling ADCs to move beyond late-line salvage settings into earlier treatment lines and combination regimens.

Importantly, early efficacy signals observed in heavily pretreated populations have repeatedly predicted later Phase III success. This paradigm is exemplified by *trastuzumab emtansine*, *brentuximab vedotin*, *trastuzumab deruxtecan*, and *sacituzumab govitecan* (e.g., NCT00829166; NCT00848926; NCT02564900; NCT01631552). This experience has driven the widespread adoption of biomarker-enriched expansion cohorts and early translational endpoints in ADC development. As a result, prioritization of promising constructs and indications has become more efficient.

The robust overall survival (OS) benefits observed in multiple Phase III-IV trials summarized in Table 2 reflect a maturation of ADC clinical development strategies rather than incremental gains in cytotoxic potency alone.^69^ Earlier-generation ADCs validated the platform in defined late-line settings. In contrast, next-generation ADCs incorporate cleavable linkers, higher drug-to-antibody ratios, and membrane-permeable payloads. These advances have enabled broader efficacy and facilitated movement into earlier lines of therapy.^88^ Notably, the clinical success of *trastuzumab deruxtecan*^49^^,^^62^^,^^63^ and *sacituzumab govitecan*^89^ demonstrated that payload class and bystander effect can expand activity beyond traditional biomarker thresholds. These findings have directly informed subsequent development programs and trial designs.^90^^,^^91^

Conversely, late-stage failures such as *trastuzumab duocarmazine* and *depatuxizumab mafodotin* underscore that antigen expression alone is insufficient to ensure clinical benefit. This limitation is particularly evident in tumors with limited drug penetration or unfavorable biology (NCT03262935; NCT02573324). These experiences underscore the importance of integrating target biology, payload mechanism, and tissue context into ADC design and clinical positioning.

Building on the survival gains achieved in Phase III-IV monotherapy trials, ADC development has increasingly shifted toward earlier disease settings and rational combination strategies. ADCs are now being evaluated in frontline, perioperative, and consolidation settings, as well as in combination with ICI and established chemotherapy backbones.^92^ These approaches are informed by late-stage clinical data demonstrating that ADC-mediated cytotoxicity can synergize with immune modulation. A prominent examples is *enfortumab vedotin* plus *pembrolizumab* in metastatic urothelial carcinoma^93^ (NCT04223856). Consistent with this paradigm, the Phase II study of *patritumab deruxtecan* in HR⁺/HER2⁻ advanced breast cancer demonstrated clinically meaningful activity in a heavily pretreated population. These results support the extension of topoisomerase I-based ADCs into biomarker-defined, earlier-line settings.^94^ At the same time, acquired resistance is emerging as a central limitation as ADCs move earlier in treatment paradigms. Mechanisms including antigen loss, altered intracellular trafficking, payload resistance, and enhanced drug efflux have been described^95^ and are increasingly being addressed via sequential ADC strategies, payload switching, and rational combinations.

The Phase III-IV clinical trials summarized in Table 2 provide robust evidence that selected ADCs achieve clinically meaningful overall survival improvements compared with standard chemotherapy, validating ADCs as survival-prolonging therapies across multiple malignancies. In solid tumors, the most consistent survival advantages have been observed with topoisomerase I inhibitor-based ADCs. In HER2-positive metastatic breast cancer, *trastuzumab deruxtecan* significantly improved overall survival (OS) compared with *trastuzumab emtansine* (median OS 29.1 vs 23.9 months) in DESTINY-Breast03^49^^,^^62^^,^^63^^,^^96^ (NCT03529110) and extended efficacy to HER2-low disease, improving OS versus chemotherapy (23.4 vs 16.8 months) in DESTINY-Breast04^84^^,^^97^ (NCT03734029). These findings redefine HER2 as a quantitative rather than binary biomarker.

Similarly, *sacituzumab govitecan* demonstrated a statistically significant OS advantage over chemotherapy in heavily pretreated hormone receptor-positive/HER2-negative metastatic breast cancer in TROPiCS-02^89^^,^^98^ (median OS 14.4 vs 11.2 months; NCT03901339). In urothelial carcinoma, *enfortumab vedotin* plus *pembrolizumab* produced one of the largest OS gains reported in a first-line solid tumor trial. Median OS was doubled compared with platinum-based chemotherapy (31.5 vs 16.1 months) in EV-302/KEYNOTE-A39^93^ (NCT04223856). These results establish an ADC-based regimen as a new frontline standard.

In hematologic malignancies, several Phase III programs demonstrate that ADCs improve long-term outcomes when incorporated into earlier treatment lines. *Polatuzumab vedotin* combined with rituximab, cyclophosphamide, doxorubicin, and prednisone (pola-R-CHP) improved progression-free survival and showed a favorable OS trend compared with rituximab, cyclophosphamide, doxorubicin, vincristine, and prednisone (R-CHOP) in previously untreated diffuse large B cell lymphoma (POLARIX;^99^ NCT03274492). In acute myeloid leukemia, multiple Phase III-IV trials incorporating *gemtuzumab ozogamicin* into frontline chemotherapy regimens reduced relapse risk and improved survival in selected patient populations (e.g., NCT00927498; NCT00372593).

Overall, across the Phase III-IV studies summarized in Table 2, multiple ADCs achieved significant improvements in overall survival versus standard chemotherapy. These findings further establish ADCs as survival-prolonging therapies in both solid tumors and hematologic malignancies when appropriately selected and positioned. The clinical development trajectory of ADCs provides important context for these outcomes. ADCs have evolved from late-line monotherapy toward earlier-line, combination-based regimens. In parallel, late-stage pipelines are increasingly dominated by DXd-based constructs directed against clinically validated antigens. Most near-term approvals are therefore expected to arise from label expansion and earlier-line repositioning rather than the introduction of entirely novel targets.^27^^,^^28^ Continued progress will depend on integrating biomarker-driven patient selection, resistance-informed sequencing strategies, and advances in ADC engineering to sustain and extend clinical benefit.

## Discussion

Despite their targeted design, objective response rates with ADCs in late-stage clinical trials typically range from 30-60%, even in biomarker-selected populations. This observation indicates that antigen expression alone is insufficient to predict durable clinical benefit. The same limitation has been consistently observed across multiple Phase III programs, including HER2-directed, TROP2-directed, and hematologic ADCs, underscoring the need for predictive biomarkers beyond target expression.^22^^,^^71^ Moreover, clinical context is an important determinant of response. Many ADC trials have been conducted in heavily pretreated populations, where advanced disease and prior therapies are associated with reduced responsiveness. Candidate determinants of response include antigen internalization and intracellular trafficking efficiency, efflux transporter expression, DNA damage response capacity and features of the tumor immune microenvironment. In parallel, emerging computational approaches integrating multi-omic tumor data, spatial profiling, and ADC-specific parameters such as target density, internalization kinetics and payload properties are beginning to enable in silico prediction of ADC efficacy and resistance. These novel approaches offer a complementary framework to guide target selection, patient stratification, and rational clinical trial design.^100^

The development of pharmacodynamic biomarkers capable of capturing early treatment effects represents an important opportunity to refine ADC deployment. Circulating tumor DNA dynamics, circulating tumor cells and immune profiling may detect response or emerging resistance earlier than conventional radiographic assessment, enabling adaptive trial designs and earlier treatment modification in non-responders.^101^^,^^102^ Such approaches are likely to become increasingly relevant as ADCs are evaluated in earlier lines of therapy, where prolonged exposure and cumulative toxicity heighten the importance of early response assessment.

As ADC efficacy improves and treatment shifts earlier in the disease course, acquired resistance is emerging as a central clinical challenge.^95^ Mechanisms described to date include antigen loss or downregulation, impaired internalization or intracellular trafficking, payload-specific resistance and enhanced drug efflux. Late-stage clinical experience supports the exploration of sequential ADC strategies hat employ alternative targets or payload classes. In parallel, rational combination regimens, particularly with ICI, are being investigated to delay or overcome resistance and extend clinical benefit.^24^^,^^103^^,^^104^

In summary, ADCs have evolved into a clinically validated therapeutic class with demonstrated overall survival benefits across solid tumors and hematologic malignancies. Continued progress will depend on improved biomarker development, resistance mitigation strategies, induction of ICD and optimized combination approaches. As these challenges are addressed, ADCs are likely to remain central to the future of precision oncology.

## Data Availability

Data sharing is not applicable to this article as no new data were created or analyzed in this study.
